# Supplementary material for: Regional Variability of Chestnut (Castanea sativa) Tolerance Toward Blight Disease
Source: Plants (Basel). 2024 Oct 31;13(21):3060. doi: 10.3390/plants13213060 (PMC11548496; doi:10.3390/plants13213060)
Supplement: Supplementary file 1 [file plants-13-03060-s001.zip › Table S2.pdf]

Table S2. Measurement data obtained in the inoculation experiment.

Population	Latitude	Longitude	Sample type
Poreč	45.244894	13.63233	Stem
Buje	45.43456	13.775746	Stem and bark
Učka	45.287679	14.247797	Stem
Cres	45.129851	14.322889	Stem and bark
Ozalj	45.578495	15.418059	Stem
Samoborsko gorje	45.829877	15.60997	Stem
Medvednica	45.870015	15.939987	Stem
Hrvatska Kostajnica	45.22018	16.494295	Bark
Moslavačka gora	45.59001	16.759987	Stem
Požega	45.310752	17.664591	Bark
