# Supplementary material for: Regional Variability of Chestnut (Castanea sativa) Tolerance Toward Blight Disease
Source: Plants (Basel). 2024 Oct 31;13(21):3060. doi: 10.3390/plants13213060 (PMC11548496; doi:10.3390/plants13213060)
Supplement: Supplementary file 1 [file plants-13-03060-s001.zip › Table S3.pdf]

Table S3. Factorial ANOVA for the effect of a particular genotype of chestnut branches (Genotype), chestnut population of origin (Population), and particular fungal isolate and viral strain combination (Inoculum), and the effect of CHV1 presence or absence in a fungal isolate used in inoculation (Inoculum type) on lesion size.

**Effect**	**SS** [a]	**Deg. of Fr.** [b]	**MS** [c]	**F** [d]	***p* Value**
Genotype	413775.9	67	6175.76	10.2100	0.000000 *
Population	16975.8	2	8487.91	14.0325	0.000001 *
Biogeography	-	0	-	-	-
Inoculum	733030.0	6	66639.09	110.1703	0.000000 *
Inoculum type	1999.5	1	1999.49	3.31393	0.069019

[a] Sum of squares, [b] Degrees of freedom, [c] Mean squares, [d] F ratio, * statistically significant values ($p<0.05$).
